# A Review of Telemedicine Interventions for Weight Loss

**DOI:** 10.1007/s12170-021-00680-w

**Published:** 2021-07-15

**Authors:** Kelsey Ufholz, Daksh Bhargava

**Affiliations:** Department of Family Medicine and Community Health, Case Western Reserve University, 11100 Euclid Avenue, Cleveland, OH 44106 USA

**Keywords:** Telemedicine, Obesity, Weight loss, Videoconferencing, Nutrition, Physical activity

## Abstract

**Purpose of Review:**

Telemedicine has become popular as an alternative for in-person weight loss treatment during the COVID-19 pandemic. This review focuses on weight loss interventions utilizing real-time telemedicine.

**Recent Findings:**

Telemedicine interventions are usually run as a weekly counseling and educational session or as a complement to a primarily Web-based intervention. A wide variety of healthcare professionals may provide the intervention. Common content includes portion control, increased physical activity, and relapse prevention. Self-monitoring is associated with intervention success. Modalities considered include online chats, text messages, phone calls, and videoconferences. Videoconferencing may be especially useful in capturing the interpersonal connection associated with in-person care but is understudied compared to other modalities. While many interventions show improvements in weight and weight-related outcomes, small sample sizes limit generalizability. Technology access and digital literacy are both necessary.

**Summary:**

Telemedicine interventions can successfully help patients with obesity lose weight. Telemedicine interventions provide a safe, remote alternative and may expand treatment access to hard-to-reach populations. Further research is needed on telemedicine weight loss treatments for seniors, men, and ethnic minorities, as well as on the impact of long-term interventions.

## Introduction: Telemedicine and Weight Loss in the Pandemic Era

The COVID-19 pandemic has been damaging for weight loss. There is some evidence that the COVID-19 has been associated with weight gain among adults [1]. This trend, a continuation of decades of elevated weight among American adults [2], illustrates the pressing need for weight loss interventions.

To provide safe care alternatives during the COVID-19 pandemic, many health systems suspended elective procedures and nonurgent outpatient visits. A large proportion of outpatients visits were converted to telemedicine appointments [3]. Telemedicine is part of a broad category of health interventions delivered via a digital health platform. Telemedicine is often used interchangeably with other digital health interventions such as virtual care, eHealth, and mHealth [4•]. Telemedicine in the USA grew an unprecedented 80% in 2020 [5]. Recent changes in legislation and reimbursement practices mean that telemedicine appointments are valued financially comparably to in-person medical appointments [6, 7]. Telemedicine also confers a number of advantages, including elimination of time spent traveling to appointments and in waiting rooms, and possibly greater ease for patients who do not need to leave their homes to seek medical care [8]. Telemedicine is expected to remain an integral part of future primary care, even after the current emergency has passed [9].

The purpose of this review is to describe published reports on telemedicine for the treatment of obesity. We defined telemedicine interventions as those involving real-time interaction with one or more weight loss professionals or peer leaders and in which counseling or other interventions are delivered remotely, with no in-person contact. Provider contact may be delivered via synchronous audio-visual videoconferencing, audio-only telephone or mobile phone contact, text messages, etc. Search terms used were “weight loss” and “weight loss maintenance,” paired with “telemedicine” or “videoconferencing,” using the search engines PubMed and GoogleScholar. Search criteria included English-language publications which described populations aged 18 or older, who were initially overweight (BMI ≥ 25 kg/m^2^) or obese (BMI ≥ 30 kg/m^2^), with publication dates 2016–2021. Reviewed studies (see Table 1) were required to have weight-related outcome data, such as changes in body weight, daily energy consumption, or daily physical activity. Other outcomes, such as exercise self-efficacy and intervention satisfaction, were also extracted if available. Entirely automated online programs not including scheduled, real-time interaction with a healthcare provider were also excluded.

**Table 1:** Summary of characteristics of telemedicine weight loss interventions

Author(s)	Date	Duration	Modality	Target population	Intervention name	Study type	Theory or model program	Active components	Outcomes
Alencar et al	2020	12 weeks	Videoconferencing+ activity tracker and scale	Adults	Telehealth Enabled Approach to Multidisciplinary care (TEAM™)	RCT with self-guided control group	None given	Weekly counseling with dietician; monthly counseling with physician; weekly weight tracking and daily activity monitoring via app	Device adherence*; rate of weight lost per week^*^
Barnason et al.	2019	12 weeks + 12-month follow-up	36 virtual Viterion sessions; handbook on portion control; 2 telephone coaching sessions	Rural cardiac rehabilitation patients	None given	RCT with cardiac-rehabilitation only control group	Social Cognitive theory/Diabetes Prevention Program	Written calorie goals; portion control; self-monitoring, self-evaluation and self-regulation	Pounds lost/BMI*; patient activation*; self-efficacy for specific eating habits; self-efficacy for situational/environmental diet management*; diet monitoring strategies*; diet planning strategies*; preparing/buying food strategies*; portion control strategies*; activity monitoring strategies*; physical activity; self-efficacy for diet management outcome expectations; cardiac exercise self-efficacy; diet social interaction strategies; diet cognitive strategies; activity social interaction strategies; activity cognitive strategies
Bradley et al.	2017	10 weeks	Online educational modules + telephone coaching	Bariatric patients exercising post-operative weight gain	Project HELP: Healthy Eating and Lifestyle Post-surgery	Pre-post feasibility trial	Acceptance-based therapy	Online interactive “skill building” modules; online quizzes; food tracking app (MyFitnessPal); track and record weight online; 20-min coaching session every 2 weeks; online discussion board	Weight loss^#^; eating disinhibition^#^; eating cognitive restraint^#^; internal responsivity to food cues^#^; emotional eating (anxiety)^#^; food-related acceptance^#^; physical activity acceptance^#^; external responsivity to food cues; emotional eating (anger and depression); food cravings; mindfulness; calorie intake; energy expenditure
Das et al.	2017	11 weeks	Videoconferencing	Worksite employees and community members	iDiet ® commercial diet program	Nonrandomized trial; virtual vs. in-person and worksite vs. community samples	None given	60-min weekly meetings; education sessions; group discussion and support; optional website message board; optional online weight logging; 500 kcal/day reduction and increased exercise; reduce hunger and food cravings; provided self-selected reduced calorie meals	Weight loss^%^
Johnson et al.	2019	12 weeks	Videoconferencing	Nondiabetic sedentary adults	None given	RCT video-conferencing vs. in-person vs. control (devices without feedback)	None given	Diet tracking (MyFitnessPal app); accelerometer watch; multidisciplinary health coaching	Steps/day*; weight loss*; insulin resistance (HOMA-IR)^#^; blood glucose; insulin; HbA1C
Marra et al.	2019	12 weeks	Combination virtual + telephone sessions with RDN	Middle-aged to older men with at least one weight-related comorbidity	None given	RCT vs. usual care control group (education materials)	None given	Individualized calorie goals; diet and weight trackers; diet educational handbook; SMART goals; medical nutritional therapy sessions	Body weight^#^; waist circumference^#^; body fat percentage^#^; calorie intake^#^; dietary quality^#^
West et al.	2019	6 months	Videoconferencing	Women	None given	RCT video vs. text-based group chats	None given	60-min weekly group meetings; education al content on restricting kcal intake and increase physical activity; self-monitoring; goal setting; problem solving; relapse prevention	Weight loss; treatment engagement*; self-monitoring of weight*; self-monitoring exercise; minutes of physical activity*; attendance; self-monitoring diet; website logins; viewed weekly lessons*; accessed and submitted activities; accessed discussion board*; posts viewed, initiated, and replied to*; total discussion board interactions*

*Greater changes compared to control

^#^Statistically significant pre-post changes

^%^Statistical significance not noted

## Prior Reviews of Digital Health Interventions for Weight Loss

Two important questions should be considered when utilizing telemedicine for weight loss. First, do telemedicine interventions lead to meaningful improvements in important weight loss metrics, such as body weight, energy intake, and physical activity? Houser et al. [10•] reviewed weight and weight-related outcomes in digital health interventions published 2010–2017. Sixty percent of reviewed studies reported statistically significant declines in weight. All the reviewed studies reported improvements in diet and physical activity, although few reached statistical significance. Alternately, a systematic review of systematic reviews by Sorgente et al. explored the efficacy of Web-based weight loss and weight loss maintenance interventions [11]. Web-based digital health interventions in which program content was delivered online with no provider contact were found to be more effective compared to control and/or minimal interventions, such as receiving a weight loss manual. Results were less definitive when comparing these Web-based interventions to telemedicine alternatives, with remote provider contact. Compared to solely Web-based interventions with no contact from program providers, face-to-face interventions, with tailored or clinician-lead feedback, led to better weight loss results [11].

## Common Content

Successful weight loss interventions, both in-person and via telemedicine, have common features. Participants learn to reduce their daily energy intake in a safe, sustainable way. Healthcare professionals provide content and skill-building sessions in areas such as making healthy food substitutions, portion control, selecting and preparing healthy food, mindful eating, reduced “grazing” [12•], relapse prevention, and problem-solving [13]. Interestingly, while most studies encourage physical activity, few include guided training/exercise sessions.

One of the most frequently taught skills is self-monitoring. Many weight loss interventions encourage participants to frequently record their weight, steps per day, exercise sessions, and/or dietary details. Self-monitoring may take many forms including paper logs, uploading food diaries into a website, taking pictures of meals, or utilizing a fitness application (“app”). Digital scales and physical activity trackers have become especially popular options, both within professionally led interventions and for self-directed programs. As of May 2018, the most popular fitness applications, such as FitBit (27.4 million) and MyFitnessPal (19.1 million), have millions of active users in the USA [14]. These devices are well-suited to remote monitoring as they allow providers to access objective, real-time data and then provide tailored feedback. For example, in the TEAM intervention, participants used a Withings Activité Pop tracker and Body+ scale to self-monitor their weight, physical activity, and nutrition. Participants then received weekly feedback from a dietician and monthly medical feedback from a physician [15].

More frequent and sustained self-monitoring is associated with improved outcomes. For example, in West et al.’s comparison of text vs. videoconference weight loss groups [13], the videoconferencing group monitored their weight (123 days vs 8 days for text; *p* < 0.001) and physical activity (55 days vs 22 days) more frequently. The group which self-monitored more frequently (videoconferencing) was twice as likely to lose 5% body weight, although a small sample size (*N* = 32) precluded statistical significance.

## Intervention Structure

Interventions have been structured in two ways. One is counseling sessions, often via videoconferencing, usually hour-long weekly meetings for 10–12 weeks. Interventions lasting longer than 6 months or those with more than 14 sessions tended to result in greater weight loss [16]. Usually during these sessions, healthcare providers present educational content and then discuss participants’ questions or challenges. This format is especially useful for group interventions allowing participants to provide each other with social support, brainstorm solutions together, and strive towards a common goal. In this format, the counseling sessions are the active component of the intervention, although they may be supplemented with other activities such as text messaging groups, online discussion boards, and online educational content available on-demand.

A second common format uses telemedicine as an adjunct or supplement to another primary intervention, most often an online program. Participants receive the bulk of the intervention online, which allows them to process the content at a time and pace of their choosing. Providers monitor progress, often via apps or remote monitoring devices, and provide feedback and support at regular intervals. The frequency of the feedback may vary; Project HELP provided 20-min telephone counseling sessions every 2 weeks [17], while the TEAM intervention provided weekly and monthly feedback [15].

The background of healthcare providers who deliver telemedicine weight loss intervention varies widely. The nebulous term “health coach” is often employed. Often, a multidisciplinary team is used, which may include primary care physicians [15], nurses [12], dietitians, psychologists, or exercise physiologists. For example, in Patel’s review of motivational interviewing interventions for weight loss [16], the providers were most often classified as “lifestyle coaches,” and also included dieticians, psychologists, and medical assistants. Given the reliance upon technology, IT staff capable of providing technical support may be welcome additions, although few interventions specifically mention them. The background of providers may be less important than their ability to form a positive, supportive relationship with participants. Krukowski et al. [18] found that during an 18-month weight loss program delivered via online group chats, the quality of patient-provider bond was significantly associated with weight loss at 6 months (*p* = 0.03) but not at 18 months (*p* = 0.53). Patient-provider bond at 6 months predicted program attendance at 6 months (*b* = 0.63, *p* = 0.04), and 6-month attendance predicted weight loss at 6 months (*b* = −0.594, *p* < 0.001), even though the total weight loss explained was small (0.04 kg).

While less common, a few telemedicine interventions utilize a peer group format, allowing trained peer facilitators to lead group education and discussion sections [18]. Peer support groups for weight loss have a number of advantages, including providing social support for isolated members, increased credibility of peer facilitators who are seen as “part of the group,” and fostering a sense of community among members [19]. This may be especially important for subpopulations who do not receive social support for weight loss among their family and friends. For example, men have rates of obesity comparable to those of women [20], but only 27% of weight loss study participants are male, and only 4% of weight loss trials are all male. Men tend to view activities such as dieting as feminine and perceive little social support for weight loss from their male friends [19]. During interviews about social support for weight loss with men who had previously undergone weight loss surgery, many reported feeling a stigma to seeking weight loss counseling, as well as isolation during overwhelmingly female weight loss support groups [21]. Many men expressed a positive experience with online social support, including online support groups and social media, suggesting that telemedicine may be an acceptable option as a weight loss experience tailored to their needs.

## Technology and Digital Health Skills

Videoconferencing is an especially promising form of telemedicine, defined as telecommunication technology which allows for two-way, real-time audio and visual communication. Videoconferencing has become more common in nonclinical areas, such as education and business, and will likely remain so. Videoconferencing offers many of the advantages of in-person programs, including face-to-face interactions and the possibility of providers giving visual demonstrations, while maintaining the practical and safety advantages of remote interventions. During interviews following a 6-month group-based videoconferencing intervention, participants reported that videoconferencing “exceeded my expectations,” and expressed appreciation for the lack of required travel, saved time and money, and fewer disruptions to their workday, as well as “reduced threat,” to be attending a group session in their own homes [22•]. Participants also reported perceived social support from their fellow group members. Almost all participants reported technical difficulty, ranging from difficulty installing the videoconferencing app (Skype Business) to unreliable Internet connections, equipment incompatibility, etc. Participants felt most comfortable if they had prior experience with videoconferencing. One participant withdrew due to lack of bandwidth. A test call prior to study implementation was helpful in troubleshooting. Participants considered the technical issues to be “annoying” and “distracting” but not detrimental to the overall experience. These results highlight the need for participants to have digital literacy skills, as well as access to affordable, reliable Internet service.

Because videoconferencing is a relatively new technology, it has been less frequently studied than older technologies such as telephone and Web-chat interventions. Patel et al.’s review [16] of remote motivational interviewing interventions found that 80% used telephones, 13% email and phone, and 7% synchronous online chats, with none utilizing videoconferencing. Programs will often utilize multiple modalities, such as text messages and videoconferencing, within the same intervention. A different review from 2019 found the most common modality was mobile phones (48%), followed by telephone and videoconferencing (22% ), eHealth (26%), and wearable devices [10•]. Mobile phone interventions were more likely to show statistically significant results than those using telephone/telemedicine or EHealth. User’s prior experience with the needed technology and technology usability tended to be critical factors in intervention success [10•]. Future reviews are needed to definitively compare the effectiveness of developing technologies.

## Efficacy of Telemedicine Interventions

The second essential question to answer is: are the health outcomes of telemedicine appointments on par with what could be achieved in an in-person format? Overall results suggest telemedicine interventions can lead to clinically meaningful weight loss. For example, West et al. [13] compared the efficacy and feasibility of a group-based weight loss intervention based upon a manualized in-person intervention. Women with obesity were randomized to either an hour-long synchronous text-based group chat or videoconference group discussion. Greater attrition was observed in the text-based group (31%) compared to the videoconferencing group (12%). The videoconferencing group lost more weight (−5.0% ± 6.0% body weight) compared to the text-based group (−3.0% ± 4.1%). Both results were not significant, possibly due to the small sample size (*N* = 24 completed the study). The authors note a moderate effect size of 0.4, which would have been statistically significant with *N* = 100 (see Table 1).

Das et al. [23] studied iDiet®, a commercial weight loss program. Participants chose an in-person (25%) or videoconferencing intervention (75%). Fifty-eight percent of those who began the program achieved a 5% weight reduction. Study completers lost a mean of 7.4% ± 3.6% body weight. Seventy-four percent of completers achieved at least 5% weight loss, although the authors do not state if this was statistically significant. Greater weight loss was achieved by those who enrolled in January–March (*p* = 0.022), older than 30 years old (2.2% difference; *p* = 0.013), and worksite participants (1.2 % difference; *p* = 0.001). Virtual participants, older participants, and those in incentivized programs were more likely to complete the program (*p* < 0.004). Program success was not impacted by gender, starting BMI, or videoconference vs in-person format. The reason for the videoconferencing format’s greater popularity was not given. Because participants chose their preferred format, a greater number of participants may have chosen videoconferencing because they found the format more enjoyable or more convenient, or had prior experience with the technology. Given the comparative popularity of the videoconferencing format, the greater completion rate of that program, and the potential for widespread dissemination, the equivalent videoconferencing vs in-person weight loss results are even more encouraging (see Table 1).

Another example is Johnson et al. [24]. Sedentary adults (*N* = 30) were randomized to either a videoconferencing, in-person, or control condition. Participants in the two active conditions received personalized feedback from a multidisciplinary team of health coaches, including dieticians, exercise physiologists, and a medical doctor. The videoconferencing group showed greater weight loss (8.23 kg ± 4.5 kg; 7.7% body weight) compared to the in-person group (3.2 kg ± 2.6 kg; 3.4% body weight) and control (2.9 kg ± 3.9 kg; 3.3% body weight) (*p* < 0.05), as well as greater daily steps (*p* = 0.03). Changes in blood glucose, insulin, and HbA1C were not significantly different across interventions. The authors suggest that the videoconferencing intervention showed results superior to the in-person module because the virtual format showed better attendance (100%) compared to the in-person format (80% attendance) (see Table 1).

Alencar et al. [15] combined videoconferencing with activity trackers in an intervention designed to promote 1–2 pounds of weight loss per week. Participants were given a digital scale for weekly weight monitoring, an activity tracker for daily activity monitoring, and content from the Telemedicine Enabled Approach to Multidisciplinary care (TEAM™) intervention. Participants were randomized to regular videoconferencing coaching or to a control group that received the devices but did not receive feedback. The videoconferencing group showed greater adherence to both the scale (92% ± 0.10% vs. 75% ± 15%, *p* < 0.05) and tracker (80% ± 0.14% vs. 49% ± 15%, *p* < 0.05), and a greater rate of weekly weight loss (mean −0.74 ± SD 1.8 kg) compared to the control group (*p* < 0.05) (Table 1).

Ventura et al. conducted a telenutrition study for 40–70-year-old men. Control participants received usual care; telenutrition participants also received weekly videoconferencing or telephone health coaching sessions. Both groups showed improvements in weight loss, body fat percentage, waist circumference, overall diet quality, and calorie intake (*p* < 0.001) [25]. The telenutrition participants were more likely to lose 5% of body weight (70.4%) compared to usual care (41.4%) (*p* = 0.035) (Table 1). In general, telemedicine interventions are associated with results comparable to other formats, but studies have been usually very small.

### Telemedicine in Bariatric Patients

Bariatric surgery is one of the most successful weight loss treatments. Many patients regain weight following surgery or do not achieve their targeted weight loss goals [26]. Lifestyle interventions help maintain the loss but only 40% of patients returning for their four annual in-person follow-up visits [27]. A review of telemedicine following bariatric surgery [28] found that 90% showed positive outcomes in weight loss and nutrition knowledge, as well as a high level of satisfaction. Most were small feasibility studies. Seventy percent used telephone modality. For example, Bradley et al. converted an in-person intervention to an online format supplemented with telephone coaching. Participants were bariatric surgery patients who had regained at least 10% of their body weight since their lowest postsurgery weight [17]. Those who completed treatment lost a significant amount of weight at program’s end (5.1% ± 5.5% body weight; 5.9 kg ± 6.5 kg, *p* = 0.01), and a small amount of additional weight at 3 months’ follow-up (0.6 ± 2.7% body weight). However, only 18% of potentially interested participants enrolled and just over half of enrollees completed the study (*N* = 20 enrolled; *N* = 11 completed).

### Weight Loss for Obesity-Derived Comorbidities

Weight loss is desirable among patients with obesity-associated illnesses such as diabetes or heart disease. An example of a successful weight control intervention for diabetes is the Diabetes Prevention Program (DPP), a lifestyle-based intervention with an impressive 58% success rate in reducing incidence of diabetes [29] and effects lasting 10 years [30]. A review and meta-analysis by Joiner et al. [31] examined the success of converting DPP to telehealth or eHealth format. Behavioral support was offered remotely via a variety of modalities including videoconferencing, email, online messaging, telephone, and a mix of telephone and online messaging. The DPP content was usually delivered via a Web-based app. Meta-analysis results found DPP with on-going behavioral support resulted in greater mean percentage weight change than a standalone DPP content without support, regardless of whether that support was delivered virtually (−4.31%, 95% CI −5.26 to −3.37%, *I*^2^= 78.4%) or in-person (−4.65%, 95% CI −6.63 to −2.67%, *I*^2^= 94.5%). The authors note considerable heterogeneity in modalities used. Future research is needed to determine the optimal method. Even when not specifically targeted, telemedicine weight management interventions can positively impact metabolic markers of diabetes. Johnson et al. [24] describe a weight loss intervention in which the videoconferencing arm showed a significant improvement in insulin sensitivity, as well as greater weight loss compared to controls.

Weight loss has been observed in a variety of patients enrolled in the DPP. In one study, for example, rural-dwelling cardiac rehabilitation patients were randomized to a 12-week telehealth cardiac rehabilitation program with additional weight management components or to receive only the cardiac rehabilitation program. Participants were provided with skill-building education sessions based upon the DPP, along with telehealth coaching sessions delivered by a research nurse at weeks 9 and 12. The weight management group lost significantly more weight (13.8 pounds, or an average of 5.9% total body weight), compared to the control group (*p* < 0.05). At 6 months, the weight management group spent almost twice as much time in moderate to vigorous physical activity (24 min/day) compared to the control group (14 min/day), although this difference was not significant. Patients in the weight management group showed greater patient activation in managing their own healthcare (*p* = 0.02), and were more likely to use weight management strategies including planning diet, monitoring diet, buying food, preparing food, and portion control [12•].

## Future Research

Telemedicine may be especially useful when it allows greater healthcare access among underserved or high-risk populations, such as the elderly population. Senior citizens account for a growing percentage of the population, and many have obesity as well as obesity-related comorbidities including hypertension, diabetes, or arthritis. Despite this, few studies have examined telemedicine weight loss interventions for seniors [32], an important research gap.

Telemedicine has been described as especially useful for expanding healthcare access in rural communities, by eliminating a lengthy commute and providing access to specialists local communities lack [32]. Unfortunately, many rural communities lack access to reliable, affordable Internet. A related difficulty is the link between digital literacy skills, such as familiarity with videoconferencing, and intervention success. Future interventions may wish to combine health-related content with technical skills training or close contact with community organizations providing low-cost Internet access.

One serious limitation of the reviewed studies is sample size and generalizability. Most studies reviewed were feasibility studies, with small sample sizes (i.e., Bradley et al., *N* = 20 enrolled, *N* = 11 completed study) [17], often further divided into two or more randomized groups. Therefore, generalizability of study results is questionable at best. Alencar et al. [15], excluded participants with both type I and type II diabetes, and “receiving treatment for a serious medical condition (i.e., cancer).” While understandable in a small-scale feasibility study, such criteria severely limit the interventions’ usefulness in the general population, a large percentage of whom seek weight loss to improve obesity-associated health conditions. As previously noted, women are overrepresented in weight loss interventions, especially younger, Caucasian women. Expansion of telemedicine to men, ethnic minorities, and older adults is needed.

The ease of access of telemedicine should make longer-term interventions possible. Despite this, most interventions do not exceed a 10–12-week duration. Because studies of longer interventions show superior results and may help prevent weight regain, future studies should examine longer durations. Greater consideration should also be given to developing technologies, especially videoconferencing and remote monitoring devices, such as activity trackers, scales, and apps. Interventions utilizing weight loss as a treatment for obesity-derived illnesses may wish to incorporate remote monitoring of illness markers, such as at-home blood pressure monitoring or daily blood glucose testing.

## Conclusion

In summary, telemedicine represents a promising modality with great potential to deliver much-needed weight loss interventions. Accumulating evidence demonstrates meaningful clinical improvements in body weight, physical activity, and nutrition. Several critical research and practice gaps remain, including optimal modality and duration and usefulness in special populations.
